# Protein-protein docking using region-based 3D Zernike descriptors

**DOI:** 10.1186/1471-2105-10-407

**Published:** 2009-12-09

**Authors:** Vishwesh Venkatraman, Yifeng D Yang, Lee Sael, Daisuke Kihara

**Affiliations:** 1 Department of Biological Sciences, Purdue University, West Lafayette, Indiana 47907, USA; 2 Department of Computer Science, Purdue University, West Lafayette, Indiana 47907, USA; 3 Markey Center for Structural Biology, Purdue University, West Lafayette, Indiana 47907, USA

## Abstract

**Background:**

Protein-protein interactions are a pivotal component of many biological processes and mediate a variety of functions. Knowing the tertiary structure of a protein complex is therefore essential for understanding the interaction mechanism. However, experimental techniques to solve the structure of the complex are often found to be difficult. To this end, computational protein-protein docking approaches can provide a useful alternative to address this issue. Prediction of docking conformations relies on methods that effectively capture shape features of the participating proteins while giving due consideration to conformational changes that may occur.

**Results:**

We present a novel protein docking algorithm based on the use of 3D Zernike descriptors as regional features of molecular shape. The key motivation of using these descriptors is their invariance to transformation, in addition to a compact representation of local surface shape characteristics. Docking decoys are generated using geometric hashing, which are then ranked by a scoring function that incorporates a buried surface area and a novel geometric complementarity term based on normals associated with the 3D Zernike shape description. Our docking algorithm was tested on both bound and unbound cases in the ZDOCK benchmark 2.0 dataset. In 74% of the bound docking predictions, our method was able to find a near-native solution (interface C-*α*RMSD ≤ 2.5 Å) within the top 1000 ranks. For unbound docking, among the 60 complexes for which our algorithm returned at least one hit, 60% of the cases were ranked within the top 2000. Comparison with existing shape-based docking algorithms shows that our method has a better performance than the others in unbound docking while remaining competitive for bound docking cases.

**Conclusion:**

We show for the first time that the 3D Zernike descriptors are adept in capturing shape complementarity at the protein-protein interface and useful for protein docking prediction. Rigorous benchmark studies show that our docking approach has a superior performance compared to existing methods.

## Background

Protein-protein interactions are a pivotal component of many biological processes and mediate a diverse variety of functions that include signal transduction, antibody-antigen complex, transport, and gene expression regulation, to name a few [1-4]. Although detailed structural information of a protein complex is necessary for the understanding of the underlying mechanisms, such details are often difficult to obtain through traditional experimental means (X-ray crystallography, NMR). To this end, a number of computational protein docking methods have been developed [5,6], which employ various schemes for describing both geometric and energetic complementarity at the docking interface and explore the docking conformational space [7-11].

Although significant efforts have been made to develop protein docking prediction methods, recent Critical Assessment of PRediction of Interactions (CAPRI) [12] experiments have shown that plenty of room for improvement still remains. It is often discussed that one of the weaknesses of current docking methods is that they are not fully capable of handling the inherent flexibility of protein chains, which induces varying degrees of conformational change upon docking [13,14]. Indeed, accounting for full flexibility of proteins is still a very challenging task for any computational method, especially for cases where the main-chain of a protein undergoes substantial conformational change (a root mean square deviation (RMSD) of a few Angstroms) upon docking. Such large fluctuations of loop regions or domain motions associated with several proteins is difficult to predict even using current molecular dynamics simulation [15] or *ab initio *protein structure prediction approaches [16-18]. Therefore, some of the earliest works on protein docking treat interacting proteins as rigid-bodies using geometrical complementarity at the docking interface to guide the search for putative binding modes [9,19,20].

The importance of effective methods of capturing geometrical information has been recently emphasized because shape complementarity forms the basis of most docking schemes, including those that accommodate a reasonable amount of conformational change. This can be attributed to the following reasons: (1) There are many protein complexes which undergo only subtle conformational change upon docking, which are practically well approximated by rigid-body docking methods [21]. Enzyme-inhibitors, an important class of protein complexes, fall into this category. (2) Flexibility of the protein can be handled to some extent by introducing a degree of softness in the surface representation [22-24] or by applying energy optimization [15] or side chain conformation search. (3) To account for chain flexibility in the docking prediction, pair-wise docking of simulated conformational ensembles of the interacting proteins can be performed, while treating each pre-determined conformation as a rigid body (cross-docking) [15]. (4) More broadly, effective protein surface shape representation has a wide variety of other applications, including structure-based function prediction [25-28], binding-ligand prediction [29,30], and fast protein database search [31,32]. Accordingly, there has been a renewed interest in shape driven docking [33-37], with more advanced descriptors being introduced. A brief overview of previous studies relating to protein shape representation and their application to docking is given below.

Connolly published pioneering works on detecting shape complementarity, the solid angle approach [38] and its improved variant called shape distributions [39]. These works attempted to distinguish regions with similar concavity but with different shapes. Proceeding along the same lines, a recently published method, Context Shapes (CS) [33] uses Boolean values to represent local shape features by applying a ray tracing scheme. Each context shape captures the local shape within a sphere centered on a surface point. The description is in the form of a binary vector with each bit assigned 1/0 depending on its location, *i.e*. inside or outside the surface. A rotational search is then performed to identify docking orientations that are ranked in terms of the buried surface area. Some approaches have also used surface normals for defining docking orientations and shape complementarity [40,41]. For example, the *S*_*C *_statistic uses the product of the normals of the points at the interface weighted by the distance between them to quantify the geometrical match [42]. In the approach proposed by Bordner and Gorin [43], surface normal vectors are anti-aligned and rotated along the common axis provided by the contacting normal. PatchDock combines geometric hashing and pose clustering to identify matching patterns across geometric patches (concave, convex, flat) in the participating surfaces [44]. The algorithm relies on the use of transformation invariant shape signatures (local reference frames) that are defined using pairs of points and their associated normals. The patch based comparison is also used in another docking program 3D-GARDEN [45] that compares the triangular facets of the ligand and receptor surfaces to define initial docking orientations. 3D Grid-based representations, on the other hand, provide a discretized form of the protein surface and volume and have been a popular choice in several docking approaches [19,23,46,47]. While minor conformational changes can be handled implicitly, the surface complementarity of the docked structures can be assessed in terms of the grid overlap, which can be evaluated by applying Fast Fourier transforms (FFT).

More recently, several publications have featured the use of spherical harmonics and its extension, the 3D Zernike descriptors (3DZDs), which have been successfully applied to comparing shapes of proteins and ligands [31,32,48,49]. HEX [50], for example, uses spherical polar basis functions to model surface shapes. It also avoids the use of expensive grid-based calculations employed in FFT based methods and instead uses the expansion coefficients of spherical harmonics to calculate correlations of the ligand and receptor surface overlaps. Spherical harmonics, however, are not rotationally invariant and make use of Wigner matrices [51] to identify rotationally invariant regions. In contrast, 3DZD corrects this drawback while providing a more compact shape definition. Our previous studies [25,31,32,52,53] have shown that the rotation invariant descriptor effectively captures protein surface shape *similarity *on both global and local levels. To our knowledge, no known application of the 3DZD to docking has been reported in literature.

In this paper, we introduce, for the first time, the application of the 3DZD [25,54] for docking. The present work uses 3DZD for capturing protein shape *complementarity *and applies it to protein-protein docking by matching local regions around points across arbitrary rotations. The docking algorithm named LZerD (Local 3D Zernike descriptor-based Docking program) LZerD uses a geometric hashing scheme [10] and incorporates a novel geometric scoring function that serves as an efficient first stage filter. The method does not use any binding site information and is moderately tolerant to conformational changes. The efficacy of the method is demonstrated using the ZDOCK benchmark datasets [21,55] and results are compared with other approaches [33,33,47,50,56]. We first show that 3DZD is able to capture shape complementary of docking interfaces effectively and by applying 3DZD to a docking scoring function it improves docking prediction results. We further show that LZerD has a better performance than others in unbound docking predictions while staying comparable for bound-bound docking cases.

## Results

### Capturing local shape complementarity using 3D Zernike descriptors

To begin with, we demonstrate that the 3DZD sufficiently captures the shape complementarity of protein docking interfaces and can also quantifies the complementarity. As explained in the Methods section, the 3DZD is a series expansion of a three dimensional (3D) function (*i.e*. in this work protein surface is represented by a 3D function). Hence, two surfaces are compared by the series of coefficients assigned to each term in the 3DZD. Here, we compute the correlation coefficient of the 3DZDs of two surfaces to quantify the similarity. A strong advantage of 3DZD is that it is rotation and translation invariant, *i.e*. the same 3DZD is obtained for an object in any pose. While our previous studies used the 3DZD for capturing *similarity *of global and local shapes [25,31,32,57], this study focuses on their use in capturing the *complementarity *across local surface regions and for protein docking prediction.

**Figure 1:**
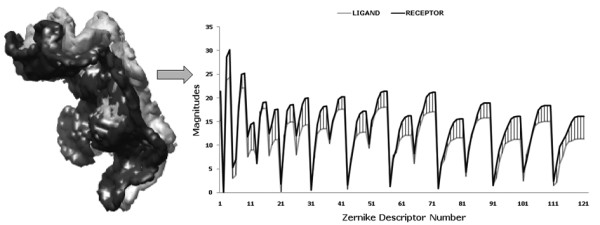
**The interface region for the protein complex **1AY7**(ribonuclease SA complex with barstar)**. Magnitudes of 3DZDs of docking interface regions of ribonuclease SA (1AY7-A, interface shape and corresponding 3DZD are shown in black) and barstar (1AY7-B, the interface is shown in white and the corresponding 3DZD is in gray). 3DZD of the order n = 20 is used. Although small discrepancies can be seen in terms of the magnitudes, the high correlation coefficient of 0.95 indicates the similarity of the regions across the interface. The difference of the 3DZDs is emphasized by striped regions.

**Figure 2:**
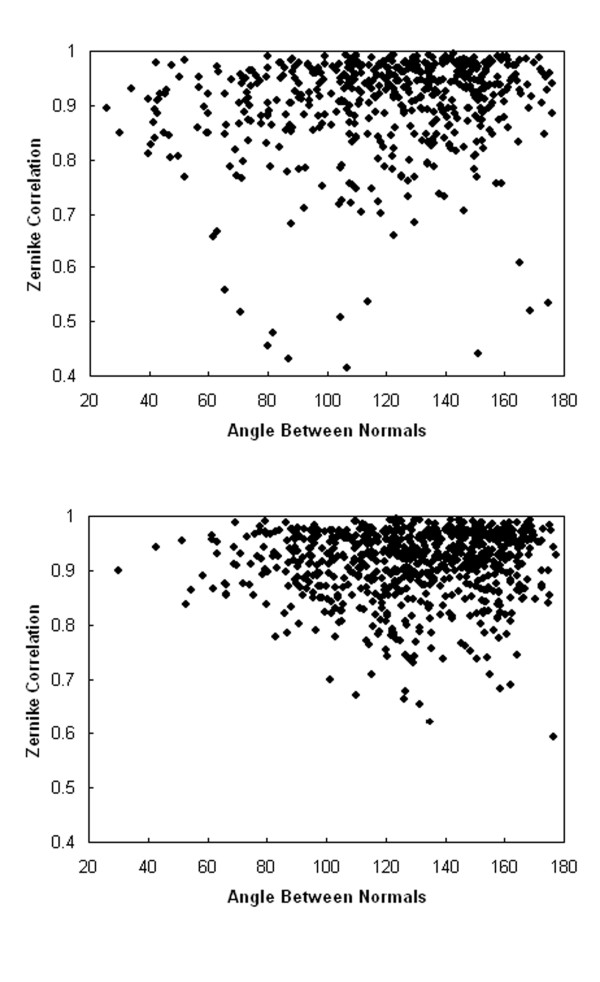
**The correlation coefficient of 3DZD and the angle between normal vectors sampled at surface points of (a), the bound and (b), unbound interface of Ras-RasGAP complex**. The PDB ID is 1WQ1 and 6A21/1WER for the bound and the unbound proteins, respectively.

The 3DZD of two objects with perfect shape complementarity are identical with a corresponding correlation coefficient of 1.0, as illustrated for a hypothetical case in the Additional file 1. Table 1 lists fifteen protein complexes, for which the 3DZDs of the docking interfaces of bound and unbound proteins are compared. Five complexes are taken from each of the three complex categories in ZDOCK benchmark 2.0, *i.e*. the enzyme/inhibitor, the antigen/antibody, and the others. Docking interfaces of both bound and unbound proteins are compared. The interface is defined as the set of surface grid points within 4.5 Å of any atom of the other protein. Also shown is the change in accessible surface areas upon the complex formation as computed by the NACCESS program [58]. Overall, a high correlation is observed among the 3DZD of the docking interface regions for both bound and unbound complexes; all fifteen cases show a correlation coefficient higher than 0.9 for the bound cases and for all but two of the unbound docking interfaces. SA-barstar complex (PDB: 1AY7) is shown in Figure 1, which has a significant 3DZD correlation of 0.93. There are five cases in Table 1 where the unbound interface shows a higher correlation coefficient than the bound interface with the difference of 0.01 or 0.02 (1ATN). To further examine this effect, we rotated the docking interface of two proteins, 1AHW and 2PCC and computed the differences in the correlation coefficients across the different orientations (taken at 30 degree intervals along the x, y and z axis). We found that these are within the error range (within 0.02) and can be attributed to the discretization of the molecular surface i.e. caused by placing proteins in different orientations on the 3D grid (the size of the grid is set to 0.6 Å). Note that the 3DZD is rotation invariant from mathematical point of view, but in practice causes errors due to the voxelization of protein surface. The effect of the variance of the 3DZD upon rotation of the protein has been examined in our previous paper (See Figure 2 in the paper by Sael *et al*. [31]).

**Table 1:** Correlation coefficient of 3DZD for bound and unbound interfaces for some protein complexes from the ZDOCK Benchmark datasets.

PDB^a)^	Class^b)^	Difficulty^c)^	#Atoms (R, L)^d)^	ΔASA (Å^2^)^e)^	3DZD Correlation for Bound interface	3DZD Correlation for Unbound interface
1AY7(1RGH;1A19)	E	E	747,721	1237	0.93	0.93
1CGI(2CGA;1HPT)	E	E	1800,441	2053	0.96	0.97
2PCC(1CCP;1YCC)	E	E	2339,847	1141	0.99	0.98
2SNI(1UBN;2CI2)	E	E	1932,521	1628	0.97	0.95
1ACB(2CGA;1EGL)	E	MD	1799,575	1544	0.94	0.95
1AHW(1FGN;1TFH)	A	E	3304,1622	1899	0.99	0.98
1FSK(1FSK;1BV1)	A	E	3347,1231	1623	0.95	0.89
1MLC(1MLB;3LZT)	A	E	3290,1000	1392	0.97	0.96
1NCA(1NCA;7NN9)	A	E	3329,3067	1953	0.99	0.99
2JEL(2JEL;1POH)	A	E	3297,640	1501	0.92	0.90
1KXP(1IJJ;1KW2)	O	E	2782,3527	3341	0.96	0.97
1IB1 (1QJB;1KUY)	O	MD	3673,1312	2808	0.95	0.87
1WQ1(6Q21;1WER)	O	MD	2534,1351	2913	0.92	0.93
1ATN(1IJJ;3DNI)	O	D	2942,2036	1774	0.95	0.97
1DE4(1A6Z;1CX8)	O	D	3064,1351	2066	0.98	0.97

a) The PDB IDs for the unbound proteins are shown in brackets.

b) The complex category labels taken from the ZDOCK benchmark 2.0. E: enzyme/inhibitor; A: antibody/antigen; O: Others.

c) The prediction difficulty level taken from the ZDOCK benchmark 2.0. E: easy (rigid-body); MD: medium difficulty; D: difficult.

d) The total number of atoms in the receptor and ligand of the unbound forms of the proteins are shown.

e) The change in accessible surface area (calculated using NACCESS) upon complex formation.

As the interface is not known in advance in blind docking predictions, the 3DZDs are computed for local spherical regions centered at selected surface points, then combinations of compatible spheres across the ligand and receptor proteins are sought. The surface normals associated with the points are also combined with the 3DZD to be able to capture both local direction and shape of surface. Figure 2 shows examples of the 3DZD correlation and the angle between the normals calculated for points less than 2.5 Å apart in the bound and unbound interfaces of the Ras-RasGAP complex (PDB: 1WQ1). As is shown later, the docking prediction for this complex turned out to be successful by using these two variables to describe the local shape features of the ligand and the receptor.

### Docking prediction results

In what follows, we report results of docking predictions by our algorithm, LZerD. To rank the predictions, LZerD uses a combination of four scoring terms, a reward and a penalty term derived from the 3DZD and normal vectors, the buried surface area, and a penalty term for atomic clashes measured in terms of the excluded volume. Docking conformation search is performed using an efficient geometric hashing scheme that incorporates a kd-tree nearest neighbor algorithm. Weighting factors for combining the four scoring terms are optimized on a training dataset which consists of 29 bound-bound protein complexes taken from ZDOCK benchmark dataset 0.0 and 1.0. The resulting scoring function is then tested on ZDOCK benchmark dataset ver. 2.0. Please refer to the Methods section for details.

First, the results for the training dataset, on which the weighting factors are optimized, are shown (Table 2). In the second part, results of the unbound docking using LZerD are reported for 84 Benchmark 2.0 complexes are compared (Table 3). Predictions by LZerD are further analyzed with respect to previously published results of other geometry based docking approaches:

**Table 2:** Comparison of the combination of the scoring terms.

Complex	LZerD	3DZD+NORMAL	NORMAL	BSA	EXVOL	EXVOL +BSA
1A0O	704	11917	5320	848	5888	**573**
1AVW	41	**26**	31	958	7412	696
1AVZ	**21**	920	554	2237	332	77
1BQL	507	6604	**294**	10042	23281	935
1BRC	**132**	221	8657	4140	2335	1456
1BRS	**1**	3	**1**	20	14154	6
1BTH	**1**	**1**	**1**	18	10176	**1**
1CHO	**1**	**1**	**1**	534	98	97
1CSE	**5**	22	18	208	16286	182
1EO8	3791	7869	6472	12640	**204**	687
1FBI	**202**	4055	4004	7480	9164	1053
1FSS	**26**	4292	3904	204	4220	85
1GLA	**1161**	42275	39867	12647	1377	4074
1IAI	**11**	45	78	895	17448	25
1IGC	2946	5887	7988	6832	2292	**1827**
1JHL	2901	**850**	1413	8706	2191	1601
1MEL	125	30081	21657	533	3405	**26**
1QFU	**17**	54	57	2571	6279	115
1SPB	**1**	63	36	107	27227	4
1STF	**2**	13	8	101	2491	3
1TAB	20	31	40	271	2878	**17**
1TGS	**1**	**1**	**1**	426	837	11
1UGH	**1**	4	**1**	5	12311	**1**
2KAI	**37**	150	337	1750	775	374
2PTC	**9**	**9**	22	1781	12777	72
2TEC	**2**	536	514	9261	289	7184
2VIR	5243	18668	15018	**3750**	5063	9594
3HHR	**351**	16048	901	1332	8496	382
4HTC	**1**	**1**	**1**	**1**	17361	**1**

**Summary**						
**Wins vs. LZerD^a)^**	**-**	3D+N/LZD 2/22	Normal/LZD 3/20	BSA/LZD 1/27	ExVol/LZD 4/25	E+B/LZD 6/20
**Wins Overall^b)^**	20	7	7	2	1	7

The training set included 29 bound-bound protein complexes taken from Benchmark datasets 0.0 and 1.0. The Rank and the iRMSD shown correspond to the best ranked hit with an interface RMSD ≤ 2.5 Å. The parameters for combining terms for the LZerD score and BSA + EXVOL are shown in Table 5. The abbreviations used are: BSA, the buried surface area term; EXVOL, the amount of clashes measured by the excluded volume; 3D+N, 3DZD+Normal; LZD, LZerD; E+B, EXVOL+BSA.

a) The number at "Wins vs LZerD" indicates the number of cases the either the ranking scheme or the LZerD shows a better rank than the other among the two scoring schemes. Ties are not counted.

b) The number at "Wins Overall" indicates the number of cases the ranking scheme reports the best rank. Wins are shown in bold in the table.

**Table 3:** Comparison for the Benchmark 2.0 unbound complexes.

	Original ZDOCK Rank			Context Shapes (CS)		PatchDock		ZDOCK Decoys Reranked by LZerD Score			LZerD		
	
Complex	Rank^a)^	iRMSD	HIT2K	Rank	iRMSD	RMSD	iRMSD	Rank	iRMSD	HIT2K	Rank	iRMSD	HIT2K
1AHW	268	2.28	21	402	2.46	181	2.49	15	1.68	50	5	1.34	42
1AK4	-^b)^	-	-	-	-	-	-	(NA	NA	NA)	43787	2.35	0
1AKJ	4872	2.29	0	-	-	-	-	1985	1.93	1	-	-	-
1AVX	2863	2.23	0	-	-	-	-	5689	2.22	0	786	2.41	2
1AY7	5584	1.33	0	-	-	-	-	394	1.1	7	1884	1.98	1
1B6C	1717	2.43	2	-	-	-	-	497	2.13	8	1001	2.41	1
1BJ1	129	0.86	49	1893	1.93	-	-	306	1.01	20	298	1.86	7
1BUH	14556	2.37	0	-	-	-	-	11230	2.42	0	12251	1.6	0
1BVK	3970	1.94	0	-	-	2754	2.27	9560	2.43	0	5515	2.24	0
1BVN	502	1.97	13	34	2.34	-	-	8	2.26	59	27	2.32	6
1CGI	145	2.44	9	-	-	1120	2.11	1775	2.14	1	9041	2.1	0
1D6R	2951	2.03	0	-	-	-	-	5022	2.49	0	2619	2.24	0
1DFJ	9	2.27	40	-	-	-	-	9350	2.14	0	-	-	-
1DQJ	2287	2.48	0	-	-	-	-	5391	2.32	0	20816	2.09	0
1E6E	22643	2.08	0	-	-	-	-	432	1.94	2	52	2.13	8
1E6J	15	1.56	34	-	-	-	-	2509	1.81	0	439	2.18	8
1E96	3094	2.26	0	-	-	-	-	882	1.88	2	216	2.14	2
1EAW	3	1.54	62	94	2.29	85	2.29	5	1.48	111	20	2.42	10
1EWY	259	2.32	2	-	-	-	-	1007	2.14	4	349	2.36	14
1EZU	1100	1.94	3	-	-	-	-	589	1.42	4	824	1.21	2
1F34	5	2.2	13	-	-	490	1.81	5082	1.61	0	-	-	-
1F51	230	2.18	4	-	-	-	-	154	1.76	5	3545	1.58	0
1FQJ	9889	2.29	0	-	-	-	-	628	2.39	2	-	-	-
1FSK	1	1.63	105	20	1.57	221	2.39	29	1.57	76	15	2.4	11
1GCQ	24339	2.29	0	-	-	-	-	39221	2.29	0	9418	1.8	0
1GHQ	-	-	-	-	-	-	-	(NA	NA	NA)	15357	1.68	0
1GRN	1704	2.34	2	-	-	-	-	1884	1.74	1	1407	2.18	1
1HE1	4672	1.31	0	1029	2.17	-	-	51	2	8	267	1.98	2
1HIA	-	-	-	-	-	-	-	(NA	NA	NA)	44189	2.42	0
1I9R	50	2.45	41	-	-	-	-	57	1.96	10	95	2.39	21
1IJK	52731	2.44	0	-	-	-	-	39460	2.44	0	6731	2.45	0
1IQD	612	2.27	5	-	-	-	-	36	0.99	27	41	1.2	18
1JPS	171	1.81	9	-	-	-	-	5305	1.37	0	292	0.9	20
1K4C	20806	1.53	0	-	-	-	-	4468	1.18	0	1188	1.43	7
1KAC	2896	2.33	0	-	-	-	-	1313	2.33	1	655	2.18	3
1KTZ	53599	1.69	0	-	-	-	-	33926	1.69	0	12162	1.19	0
1KXP	1734	2.36	1	-	-	-	-	32023	1.91	0	14208	2.22	0
1KXQ	212	1.91	13	2226	1.73	-	-	629	1.24	4	73	1.68	14
1MAH	92	1.31	9	597	1.16	887	2.28	541	0.89	6	92	0.87	2
1ML0	36	1.56	21	-	-	231	2.02	406	1.37	6	559	2.38	3
1MLC	110	1.19	12	18	2.28	-	-	243	1.07	12	1834	1.16	1
1NCA	14	1.93	47	-	-	-	-	302	1.55	12	12528	1.5	0
1NSN	185	1.81	5	26	1.79	-	-	147	1.81	13	945	2.29	1
1PPE	1	0.57	218	2	2.31	-	-	1	0.72	194	1	0.83	68
1QA9	5672	1.88	0	-	-	-	-	5924	1.82	0	1381	2.19	3
1QFW	257	1.14	7	597	1.73	-	-	136	2.31	17	108	1.24	4
1RLB	-	-	-	-	-	-	-	(NA	NA	NA)	46073	1.24	0
1TMQ	314	1.88	11	783	1.68	1	1.96	90	1.45	19	50	1.45	5
1UDI	258	2.17	4	2649	2.14	27	2.42	219	2.39	3	59	2.36	6
1VFB	2734	1.79	0	228	2.46	-	-	1534	1.61	1	1303	1.69	1
1WEJ	465	2.37	8	-	-	-	-	916	1.97	1	3914	2.06	6
1WQ1	1101	2.49	2	-	-	-	-	284	2.05	2	141	1.87	2
2JEL	45	1.79	33	-	-	-	-	149	2.44	19	133	2.49	9
2MTA	-	-	-	-	-	515	2.19	(NA	NA	NA)	606	1.64	11
2PCC	-	-	-	-	-	-	-	(NA	NA	NA)	4542	2.31	0
2QFW	832	2.29	3	33	2.32	-	-	42	1.99	17	68	1.55	29
2SIC	173	1.86	24	1077	2.28	-	-	17	1.85	61	12	2.04	9
2SNI	17906	2.44	0	-	-	-	-	428	2.33	2	-	-	-
7CEI	106	1.97	24	2290	1.9	366	1.07	705	1.57	7	6765	2.03	0

**Summary^c)^**													
		ZDOCK		CS		PatchDock (PD)		LZerD Rerank			LZerD		
**Rank<100**		11		7		3		11			14		
**Rank<500**		26		9		6		26			23		
**Rank<1000**		29		12		9		32			29		
**Rank<2000**		33		15		10		38			36		
**Wins vs. LZerD Rerank**		ZDOCK/LZerD Rerank 26/26		CS/LZerD Rerank 5/34		PD/LZerD Rerank 7/34		-			-		
**Wins vs. LZerD**		ZDOCK/LZerD 24/33		CS/LZerD 5/34		PD/LZerD 8/34		LZerD Rerank/LZerD 24/28			-		

LZerD results are compared with ZDOCK, Context Shapes, and PatchDock. ZDOCK results are based on the decoy structures downloaded from the ZDOCK benchmark website. Among the 84 unbound complexes in the ZDOCK Benchmark 2.0, 60 complexes are included in this table. The rest of the 23 complexes are not included as neither ZDOCK nor LZerD produced any hits within the top 54000 predictions. Another complex (2VIS) has also been excluded as LZerD needed more memory than our computer could provide. Data for Context Shapes and PatchDock are taken from the previously published paper of the Context Shapes (Table 4 in the paper by Shentu *et al*.). The number of hits within top 2000 (HIT2K) are not provided for Context Shapes and PatchDock because the data are not shown in their paper. Also note that hits ranked over 3600 are not provided by their paper.

a) "-" indicates that the algorithm was unable to find a hit among the top 54000 predictions. "NA" in the block "Reranked by LZerD score" indicates that no hits were found in the ZDOCK decoy set analyzed.

b) The Ranks column shows the best rank of hits (predictions with an interface RMSD of less than 2.5 Å). iRMSD shows the interface RMSD of the best ranked hit. HITS2K is the number of hits found in the top 2000 ranked predictions.

c) Summary shows the number of cases where the best rank below 100, 500, 1000, and 2000 are obtained. Wins vs. LZerD indicates the number of cases in which either the algorithm or LZerD has a better rank. In comparison with Context Shapes and PatchDock, hits above the rank 3600 are not considered for LZerD since hits over 3600 are not provided for Context Shapes and PatchDock in the paper by Shentu *et al*.

1) ZDOCK(PSC) [47] - The FFT based algorithm incorporates a pairwise shape complementarity (PSC) term that takes into consideration close atomic contacts (within a distance cutoff) between the ligand and the receptor with an added penalty term to account for clashes.

2) PatchDock [56] - Geometric hashing based scheme with a grid based scoring function.

3) Context Shapes [33] - Local shape representation in terms of binary values and a scoring function based on the buried surface area.

4) HEX [50] - Uses spherical polar Fourier correlations to perform a six degree search with a corresponding steric complementarity score (Table 4).

**Table 4:** Comparison with HEX on unbound cases of Benchmark 2.0.

		HEX		ZDOCK			LZerD		
Complex	Rank	lRMSD	HITS2K	Rank	lRMSD	HITS2K	Rank	lRMSD	HITS2K
1ACB	694	8.3	3	185	9.98	5	**21**	9.98	2
1AHW	234	8	3	34	9.86	20	**5**	2.68	43
1AKJ	**209**	9.6	10	-	-	-	-	-	-
1AVX	**108**	8.9	7	604	9.43	4	-	-	-
1AY7	645	9.9	4	**568**	9.39	11	1884	6.13	1
1B6C	593	9	2	182	5.6	10	**73**	6.2	10
1BUH	743	7.7	2	-	-	-	**599**	9.73	1
1BVK	-	-	-	**70**	7.56		1125	9.83	10
1BVN	63	9.1	20	29	8.65	52	**2**	6.89	49
1CGI	**42**	9.4	17	145	3.88	32	86	8.13	12
1D6R	447	7.7	1	**303**	8.44	3	344	7.84	8
1DE4	**946**	8.6	1	-	-	-	-	-	-
1DFJ	17	9.5	14	**5**	6.64	67	-	-	-
1DQJ	-	-	-	**152**	9.82	23	-	-	-
1E6E	109	5.6	10	-	-	-	**52**	4.49	10
1E6J	-	-	-	**12**	5.34	93	87	9.97	20
1E96	-	-	-	-	-	-	**1375**	8.91	1
1EAW	9	5	20	**3**	5.43	87	6	9.95	19
1EER	**609**	9.2	8	-	-	-	-	-	-
1EWY	76	9.1	12	**22**	8.08	51	103	9.91	110
1EZU	-	-	-	-	-	-	**815**	7.89	3
1F34	124	6.7	11	**5**	5.45	20	-	-	-
1F51	**371**	9.6	5	602	9.78	4	1101	8.31	1
1FQJ	**41**	8	12	-	-	-	1014	9.63	3
1FSK	5	1.8	16	**1**	4.04	149	15	6.23	28
1GHQ	-	-	-	-	-	-	**1571**	9.14	1
1GRN	**914**	9.1	2	1704	5.81	2	1407	7.41	2
1HE1	37	6.4	18	**23**	8.14	8	47	6.2	7
1HIA	51	8.7	6	-	-	-	**1**	9.49	74
1I4D	-	-	-	-	-	-	**286**	9.11	2
1I9R	**82**	2.1	8	104	9.07	16	104	9.41	10
1IJK	**1012**	8.7	3	-	-	-	-	-	-
1IQD	-	-	-	492	8.99	11	**41**	6.46	27
1JPS	-	-	-	**171**	8.51	7	292	2.01	20
1K4C	**21**	9.6	1	-	-	-	219	9.78	6
1KAC	687	8.7	1	-	-	-	**655**	3.95	3
1KXP	**36**	9.4	13	1616	7.11	2	1226	8.05	1
1KXQ	488	7.1	5	116	7.58	29	**73**	4.33	16
1M10	**514**	9.5	2	-	-	-	-	-	-
1MAH	**2**	1.2	20	92	3.86	9	92	2.43	2
1ML0	-	-	-	**36**	2.87	35	121	5.71	2
1MLC	408	3.6	2	**110**	6.17	12	1834	4.48	1
1NCA	116	1.2	5	**14**	7.08	49	270	9.97	2
1NSN	142	1.5	6	185	5.07	19	**94**	8.61	4
1PPE	2	9.7	47	**1**	0.86	358	**1**	2.26	184
1QA9	-	-	-	-	-	-	**546**	8.07	6
1QFW	-	-	-	257	8.63	4	**108**	9.54	2
1TMQ	356	5.9	9	314	6.12	11	**50**	3.71	11
1UDI	**8**	6.2	9	32	8.04	34	19	6.4	16
1VFB	-	-	-	**22**	8.52	65	150	8.7	22
1WEJ	-	-	-	**81**	8.36	42	156	9.82	18
1WQ1	125	7.1	10	610	9.9	5	**32**	9.79	11
2BTF	-	-	-	**553**	6.39	3	-	-	-
2JEL	164	6	3	**45**	4.49	86	66	6.81	35
2MTA	136	9	4	-	-	-	**4**	7.57	48
2QFW	-	-	-	-	-	-	**68**	9.01	11
2SIC	**57**	8.8	8	173	8.62	18	127	4.59	6
2SNI	**256**	9.6	7	534	9.69	4	-	-	-
7CEI	**61**	8.7	5	106	7.11	28	-	-	-

**Summary**									
		**HEX**		**ZDOCK**			**LZerD**		
**Mean**		206		173			164		
**Rank<100**		17		19			22		
**Rank<500**		32		33			34		
**Rank<1000**		42		39			38		
**Rank<2000**		43		41			47		
**Win**		18		20			22		

The results of HEX are taken from the columns of "U-U shape-only Blind search" in Table 3 of the paper by Ritchie *et al*. (2008). ZDOCK results are taken from the decoy set downloaded. Among the 84 test cases, complexes for which none of the three methods produced a hit i.e. ligand RMSD (lRMSD) less than 10 Å are excluded from the table. In this table the ligand RMSD is used to indicate the quality of predictions to be consistent with the HEX docking results. Cases where either docking algorithm obtains a better rank are highlighted in bold. "-" indicates that the algorithm was unable to find a hit among the top 2000 predictions. Mean in summary shows the MLR score (Eqn. 16).

### Prediction results on the training set

Table 2 shows the performance of LZerD for the bound-bound cases used in the training set. A near-native structure or a hit is defined as one with an interface RMSD (iRMSD) of 2.5 Å or lower (better) to the native complex. For the 29 cases considered, a hit for 18 complexes are ranked within the top 100. Table 2 also details the effectiveness of adding the scoring terms. The first column shows the results the combined weighted score (the LZerD score) as defined in Eqn. 11 with weights as shown in Table 5 in Methods, the second uses both 3DZD and normal vectors (Eqn. 6), and the last uses only the angle between the normals. In addition, we also compare results by using the buried surface area (Eqn. 7), the excluded volume term (Eqn. 8), and the combination of the two (the last column in Table 2). The weights for combining the buried surface area and the excluded volume terms are optimized in the same way as the weights for the full LZerD score on the same dataset (Table 5). Comparison of the performance shows that the full LZerD score obtains better (*i.e*. lower) ranks in more cases (20 cases) than the combination of the buried surface area and the excluded volume terms (6 cases), indicating the usefulness of the 3DZD term. The combination of the four terms to yield the LZerD score is justified by the result that it produces the top ranked hits in 20 cases.

**Table 5:** Parameters computed for the four scoring terms.

Number of Terms	Parameter type	3DZD-Normal Reward	3DZD-Normal Penalty	Buried Surface Area	Excluded Volume
4	Weight	2.39	15.85	-0.33	0.97
	Offset value	12.81	30.68	2525.13	751.44

2	Weight	-	-	0.653	-3.43
	Offset value	-	-	928.7	69.52

The parameters, the weights and offset values are calculated based on the training set of 29 bound-bound protein complexes for the four terms (Eqns. 6, 7 & 8) and for the two terms (Eqns. 7 & 8). Results for individual protein complexes are shown in Table 2.

### Comparison with ZDOCK, Context Shapes, and PatchDock

To assess the performance of the algorithm, LZerD results were compared with those of ZDOCK(PSC) [47], Context Shapes (CS) [33], and PatchDock [56]. Comparison was performed on the bound-bound docking test set (Additional file 2) and also on the unbound test set of ZDOCK Benchmark 2.0 (Table 3). Results on the bound test set for CS, ZDOCK (PSC), and PatchDock are taken from the original article of CS [33] (Table 2 in their article). As in their study, only the top ranking 3600 predictions are considered. As for the unbound test set, ZDOCK decoys (produced by running ZDOCK in the dense mode with a 6° sampling) for the Benchmark 2.0 dataset (84 complexes) was downloaded from the ZDOCK website http://zlab.bu.edu/zdock/benchmark.shtml. For LZerD and ZDOCK, the top ranking 54000 conformations are considered. The two algorithms are compared in two ways (Table 3). First, the re-ranking performance of the LZerD scoring function, when applied to the ZDOCK decoys, is examined. Next, the docking predictions by LZerD are compared with the ZDOCK results to assess the conformation searching capability and scoring performance. Results for CS and PatchDock are taken from the original article of CS [33] (Table 4 in their article).

For the docking predictions of 76 bound-bound cases (Additional file 3), CS has a better performance than others at each rank cutoff of 100, 500, 1000, and 2000. LZerD comes a close second followed by ZDOCK and PatchDock. This order is also gauged by the number of "wins" and the MLR values (Eqn. 16), which yields 20, 56, 96 and 115 for CS, LZerD, ZDOCK, and PatchDock, respectively.

For the unbound test set (Table 3), ZDOCK and LZerD show significantly better performance than CS and PatchDock. It would seem that CS is better tuned for bound-bound docking but is not as well suited for handling unbound cases. In comparing the re-ranked ZDOCK decoys the LZerD score (the second block from the right in Table 3) with the original ranking produced by ZDOCK (the block on the left), similar success rates are seen for ranks below 500. They also have the same number of wins, *i.e*. cases when either the LZerD score or ZDOCK (PSC) has a better rank than the other. However, the LZerD score performs better when ranks above 500 are considered with as many as 32 and 38 cases in the top 1000 and 2000, respectively, as compared with 29 and 33 for ZDOCK. Comparison between the docking predictions by LZerD (the right block) and ZDOCK shows that the former has a better performance. LZerD obtains a better rank in nine more cases (24 vs. 33) and has more successful cases in the top 100 and 2000 and a tied performance for ranks below 1000. The two last rows of Table 3 show head-to-head comparison between the LZerD Rerank or LZerD with the other methods. These comparisons clearly indicate the superior performance of LZerD Reranking and LZerD Docking over the others. The head-to-head comparison between LZerD and LZerD Rerank shows that the former is better than the latter, implying LZerD's better conformation sampling scheme.

**Figure 3:**
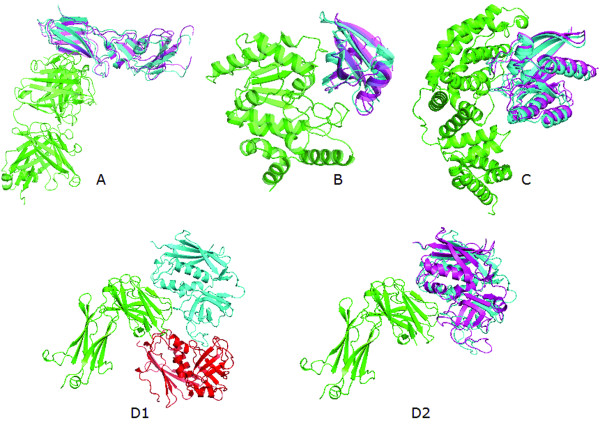
**Near-native docking configurations identified by LZerD for unbound-unbound complexes**. Top ranking hits are shown for **A**, tissue factor with inhibitory Fab. The PDB ID of bound complex: 1AHW; unbound structures: 1FGN_LH (RMSD to bound structure: 1.25 Å) & 1TFH_A(RMSD: 0.75 Å). iRMSD of the prediction shown is 1.34 Å, which is ranked fifth. **B**, uracil-DNA glycosylase with uracil glycosylase inhibitor protein complex. Bound: 1UDI. Unbound structures: 1UDH (RMSD to bound: 0.47 Å) & 2UGI_B (RMSD: 0.88 Å). iRMSD: 2.36 Å, Rank: 59. **C**, Ras-RasGAP complex. Bound: 1WQ1. Unbound structures: 6Q21_D (RMSD: 0.79 Å) &1WER (RMSD: 0.85 Å). iRMSD: 1.87 Å; Rank: 141. **D**. T-cell receptor *β *chain with superantigen SEB. Bound: 1SBB. Unbound: 1BEC (RMSD: 0.50 Å) & 1SE4 (RMSD: 0.83 Å). **D1 **is the best prediction within the top 54000 rank, which has an iRMSD of 10.24 Å. **D2 **is a hit with an iRMSD: 2.13 Å found at the rank 81598. The unbound receptor and ligand complex are shown in green and pale blue while the predicted ligand is shown in magenta.

Figure 3 shows examples of complex predictions by LZerD for four cases. 1AHW is a relatively easy case, where both LZerD and ZDOCK (PSC) have more than 20 hits in the top 2000 decoys (Figure 3A). LZerD obtains a hit ranked in the top five and significantly improves the ranking of corresponding ZDOCK decoys from 268 to 15. 1UDI (Figure 3B) and 1WQ1 (Figure 3C) are relatively difficult cases, where only a few hits are obtained within 2000 ranks by the two docking algorithms. The last case, 1SBB, is a difficult target, where both LZerD and ZDOCK failed to obtain any hits within the top 54000 ranks. LZerD generates a correct hit with an iRMSD of 2.5 Å at the rank 81598 (Figure 3D2), but the best prediction within top 54000 has an iRMSD of 10.24 Å (Figure 3D1). Therefore, the problem of LZerD for this case is the scoring function rather than the conformation sampling.

### Coparison with HEX

Table 4 shows the comparison between the results for LZerD with another shape-based docking algorithm, HEX [50] on unbound cases of Benchmark 2.0. The docking results for HEX are taken from Table 3 of the paper by Ritchie *et al*. [50]. In keeping with these results, we computed the ligand RMSD (lRMSD) and the MLR value, and excluded complexes for which no hit (lRMSD of less than 10 Å) was found within the top 2000 predictions. The results for ZDOCK are the same as in Table 3 (decoys downloaded from ZDOCK Benchmark website were analyzed).

Out of the 84 benchmark cases, all three methods failed in 25. In the remaining 59 cases, LZerD obtains a better rank in 22 while HEX and ZDOCK have corresponding values of 18 and 20, respectively. LZerD also manages to obtain the lowest MLR score of 164 as compared to ZDOCK and HEX. LZerD also outperforms the other two methods in terms of the number of cases with hits ranked within the top 100, 500, and 2000 decoys.

## Discussion

In this paper, we have proposed a novel protein docking algorithm, LZerD that employs the 3DZD for the first time for capturing protein surface shape complementarity of docking interfaces. Previously we have shown that the 3DZD is a powerful tool for identifying both global [31,32] and local [25,32] surface *similarity *among proteins. In this work, we have further shown that 3DZD is also useful for capturing protein local surface *complementarity*, which is readily applicable for protein docking. LZerD uses geometric hashing as the core of the search algorithm and takes advantage of the rotation-invariant characteristics of the 3DZD. Our experiments show that the descriptor is effective in improving docking predictions when considered as one of the terms in the scoring function.

The results on the ZDOCK benchmark datasets have been compared with the other available shape-based docking algorithms, ZDOCK, Context Shapes, PatchDock, and HEX. On the bound benchmark dataset, LZerD showed a better performance than the two methods (ZDOCK and PatchDock) but slightly worse than Context Shapes (Additional file 2). However, on the unbound benchmark dataset (Tables 3 &4), LZerD clearly outperforms all the other approaches. Hits obtained by Context Shapes, which performed well for the bound docking set, decreased sharply for the unbound benchmark dataset. In contrast, LZerD improves its performance relative to the others for the unbound cases indicating that the 3DZD is effective in handling a certain range of the flexibility of the protein surface that needs to be considered in protein docking. Shape-based approaches which can effectively handle a certain range of flexibility (an RMSD of 1-2 Å) is very important in the computational docking problem because such method is an indispensable component in a flexible docking prediction in combination with explicit conformational sampling scheme, such as "*ab initio*" structure modeling methods (*e.g*. ROSETTA [59], TASSER [17], CABS [60], TOUCHSTONE [16],) and molecular dynamics approaches [61,62], that are aimed at handling alternative conformation explicitly. Since even the state-of-the-art *ab initio *modeling methods are still not capable of precise modeling of protein structures, it is docking methods' task to be able to handle the small range of flexibility.

While the current study only uses geometric terms for scoring, the inclusion of more interaction energy based terms such as desolvation and electrostatics are expected to make further improvement. As a future work, we are following a cross docking approach using LZerD based on an ensemble of simulated structures of individual proteins to further consider flexibility of protein chains.

## Conclusion

We showed that the 3D Zernike descriptors are effective in capturing shape complementarity at the protein-protein docking interface. Employing the 3D Zernike descriptors, we developed a novel shape-based protein docking algorithm, LZerD. Comparison with existing shape-based docking algorithms showed that LZerD has a better performance than the other existing methods in unbound docking while remaining competitive for bound docking cases.

## Methods

### Protein surface representation

**Figure 4:**
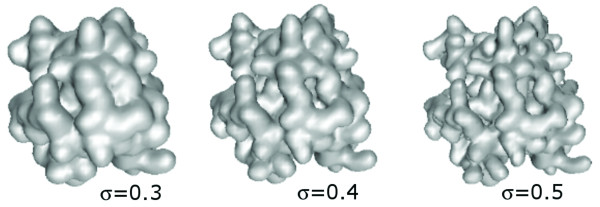
**Molecular surfaces shown for varying degrees of smoothness *σ***. Larger values reveal more detail while those at the lower end produce more spherical surfaces. The parameter *σ *is set to 0.3, 0.4, and 0.5 from left to right.

The protein surface is defined implicitly as the sum of atom-centered Gaussians [43] and takes the form of *G*(*x*) = *σ*:

where *x *is a point on the surface in the three dimensional space and *x*_*i *_the center of an atom in the protein. The degree of smoothness of the resulting isosurface is altered by varying the value of the parameter *σ*, the effect of which can be seen in Figure 4. The molecular surface definition of the form of Eqn. 1 has been commonly used for representing drug molecules [63], protein-protein interaction [64], and docking [65]. The smoothing parameter has the advantage of allowing a certain amount of flexibility to be incorporated into the representation which for this study is set to 0.35. 0.35 provides sufficient amount of surface detail and being simpler is faster to calculate as compared to the other forms proposed [64]. We used the Marching Cubes algorithm as implemented in the GNU Triangulated Surface (GTS) library http://gts.sourceforge.net to compute the protein surface, which is then discretized by placing it inside a cubic grid. Each grid cell (voxel) is then assigned 1 if it is on the surface and 0, otherwise.

### Shape representation using 3D Zernike descriptors

The protein surface defined in Cartesian coordinates is represented by the 3D Zernike descriptors (3DZD). The 3DZD are a series expansion of a 3D function, *f(x)*, (*i.e*. protein surface represented by Eqn. 1 in our case), which allows a compact representation of a 3D function. Desirable properties of 3DZD such as transformation invariance and minimum information redundancy (orthonormality) make them well suited for shape matching. Here we provide a brief description of the 3DZD. For more mathematical details, please refer to the paper by Canterakis [66] and also refer to our previous papers for technical procedure for computing the 3DZD for proteins [31,32].

A protein surface is represented as a 3D function, *f(x)*, by first placing the protein structure onto a 3D grid, and voxels overlapping with the protein surface are marked with a value of 1 and others are with 0. The size of the grid is set to 0.6 Å. The 3DZD are derived from the Zernike-Canterakis polynomials[66]. For the order *n *and repetition *m*, they are given by

where (*ϑ*, *φ*) are the spherical harmonics[67] (*(ϑ*, *φ*) are the polar coordinates) of the *l*^*th *^degree with *l *≤ *n*, *m *∈ [-*l*, *l*], and *(n-l) *is even and non-negative. The radial polynomial *R*_*nl*_(*r*) where *r *is the radius is defined so that  are orthonormal polynomials when written in Cartesian coordinates. For a 3D function *f*(*x*) where *x *∈ ℜ^3 ^the 3D Zernike moments are given by

The above equation is expressed as a linear combination of geometric moments of order *n *where *M*_*rst *_denotes the geometrical moment of the object normalized to fit in the unit sphere and  is a set of complex coefficients. The moments are however not rotationally invariant. They are therefore collected into (*2l+1)-*dimensional vectors and the norms (||·||) of the vectors  define the rotationally invariant 3D Zernike descriptors (Eqn. 4).

We use n = 10, which yields a total of 36 invariants for the 3DZD. Matching local shapes is thus reduced to comparing the vector of numbers associated with each local surface. The strength of complementarity between two such vectors (*p *= 36 values in vectors *x *and *y*) is given by the correlation coefficient:

During the docking process, 3DZD are computed for spherical patches of 6 Å radius centered on a set of evenly distributed points on the protein surface.

### Overview of docking algorithm

**Figure 5:**
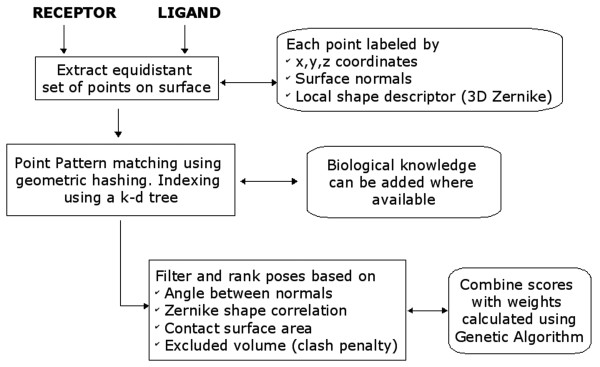
**Outline of the docking algorithm, LZerD**. Although biological knowledge of docking interface regions can be used, we have not used such additional information in this study.

A general outline of the docking procedure, LZerD, is shown in Figure 5. Starting with the atomic coordinates of a ligand protein and a receptor protein, evenly distributed points at a minimum separation of 1.8 Å are extracted from the corresponding protein surfaces. For each point, a corresponding normal vector is calculated. In all, each point is labeled by its coordinates in the 3D space, the surface normal, and a numeric vector of 3DZD that captures shape features in the region bounded by a sphere of set radius. Geometric hashing is then used to find partial matches between the two surfaces based on the signatures associated with the points. The alignment transformations calculated for the matching point sets are then applied to the ligand protein, following which, the scoring function examines the amount of overlap (with an added penalty for atom collisions). Another term that measures the local geometric complementarity as reflected by the angle between the normals and the shape correlation of the 3DZD vector is also calculated. The terms are then combined with appropriate weighting factors (determined using a genetic algorithm) to give a final score for each orientation. The individual components of the docking algorithm are described in detail in the following sections.

### Geometric hashing based point matching

Given two protein surfaces (a ligand protein and a receptor protein) represented by a set of points with corresponding labels, the first task is to quickly identify matches between the two sets of features. We have used geometric hashing [10], a computer vision based approach, for identifying candidate matches. The algorithm proceeds in two stages. In the preprocessing (first) stage, points from the ligand protein are stored in a transformation invariant form in a hash table. For this, an orthonormal coordinate frame is defined using three non-collinear points and the coordinates of all the other points are expressed in this local frame. The calculated invariant vectors are then discretized and stored in a two-dimensional hash table. In the recognition stage, points from the receptor protein are used to calculate candidate frames. For each such coordinate basis and the receptor points encoded in it, corresponding ligand points are identified (those points must satisfy geometrical constraints) in the hash table constructed earlier.

A naïve implementation of the hash table based indexing is however found to be inefficient due to the non-uniform distribution of the data in the hash space. This results in longer search times as hash entry lists have different lengths. Efforts to tackle this problem have attempted the use of rehashing techniques [68] and self-organizing maps [69]. Here, a commonly used data structure for nearest neighbor search, the *k*d-tree (*k *is the dimension), which partitions point sets recursively into a small number of cells with no cell containing too many objects, is employed. Unlike hashing, the method is adaptive in that, the partitions are dependent on the data points to be stored. We have used the approximate nearest neighbor (ANN) library, which, for a given query point, retrieves all neighbors within a specified radius and allows more efficient search than conventional kd-tree [70] approaches. These points are further checked for compatibility criteria before they are added to the pool of matches.

**Figure 6:**
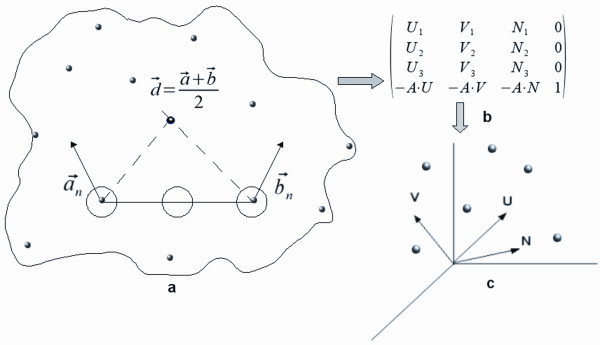
**A coordinate frame is calculated using points A and B and the average of their normals  as shown in (a)**. The change of coordinates from (X, Y, Z)-space to (U, V, N)-space is given by the rotation matrix (U, V, N) (the 3 × 3 matrix represented in the left upper corner in the 4 × 4 matrix in (**b**)) and the translation vector to the origin (assuming the point A is taken as the origin, (-AU, -AV, -AN) shown at the bottom of the 4 × 4 matrix is the translation vector). Coordinates of point C can be expressed in the new coordinate system by using a matrix vector multiplication.

Given *N*_*L *_and *N*_*R *_points on the ligand and receptor proteins, respectively, the geometric hashing scheme is as follows:

Define a reference frame using two points, *A *and *B*, and a direction  where  and  0 are the associated normals. For building the Cartesian frame, point *A *is taken as the origin,  as the *x*-axis,  as the *y*-axis and the *z*-axis as the cross-product  of the other two axes. The orthonormal basis (transformation matrix) so formed includes a rotation and a translation to move a given point to the origin as shown in Figure 6. In choosing a frame, certain constraints are applied. Most values were taken from a previously published study [9], except for two values, d_min _and d_max_, which are described below. Values ranging from 2.5 to 5 Å for d_min _and 7.5 to 12 Å for d_max _were tested on the training set proteins. Considering the number of hits and the computational efficiency, we chose 4 Å for d_min _and 9 Å for d_max_.

i. *d*_*min *_<*d*_*Ab *_<*d*_*max *_where *d*_*min *_and *d*_*max *_are the minimum and maximum allowable distances between two point are set to *d*_*min *_= 4 Å and *d*_*max *_= 9 Å

ii. *TorsionAng le*(*a*_*n*_, *A*, *B*, *b*_*n*_) < 1.4 *radians*

iii. ∠(*a*_*n*_, *b*_*n*_)< 1.2 *radians *where ∠denotes the angle

iv. 0.87 *radians *< ∠(*a*_*n*_, *B*), ∠(*b*_*n*_, *B*) < 1.2 *radians *and |∠(*a*_*n*_, *B*) - ∠(*b*_*n*_, *B*)|< 1.2 *radians *(1.4, 1.2, and 0.87 radians are approximately equivalent to 80.2, 68.8, and 49.8 degrees)

1) The coordinates of all other points in the ligand are then recorded in this reference frame created in the previous step. Thus, for a ligand defined by *P *points, the other *P-2 *points (two points used for the construction of the frame) are transformed into this reference frame. Only those points *C *for which *d*_*AC*_, *d*_*BC *_< 15 Å are considered. The triangle (*A, B, C*) thus formed is weighted by the edge lengths along with the other labels associated with the vertices.

2) Enter all the transformed points of the ligand protein in each ligand reference frame into the *k*d- tree where *k *= 3 *i.e. x, y, z *coordinates of the ligand point. Each such point has a reference to the coordinate frame in which it was transformed.

3) Compute the coordinate frames and new positions of the receptor points as shown in Steps 1 and 2.

4) For every coordinate frame for the receptor protein:

i. For every transformed point in the current receptor frame

1. Perform a range search to retrieve geometrically similar points of the ligand (points recorded in different reference frames are stored in the kd-tree). This search (using methods in the ANN library) locates all nearest neighbors of the current receptor point within a radius bound of 2.5 Å.

2. For each ligand point located within this bound, compare the labels (3DZD, normals, torsion angles, and point distances) of the points (receptor vs ligand) and those of the corresponding reference frames. If the labels are compatible, then increment a vote counter for the current ligand-receptor basis pair. Criteria for comparison are as follows:

a) The lengths of the triangle edges should be compatible *i.e*.

where *r*_*i*_, *r*_*j*_, *l*_*i*_, and *l*_*j *_are vertices of the triangles and *i, j *= 1, 2, 3.

b) A ligand point *i *is compatible with a receptor point *j *if the correlation between their 3DZD exceed a threshold value *i.e. Correlations(Z*_*i*_, *Z*_*j*_) ≥ 0.65

c) Compatibility of pairwise normal angles formed between any two of the ligand or the receptor  < 0.8 *radian *for 1 ≤ *i*, *j *≤ 3

d) Torsion angles formed by any two points and their corresponding normals should be compatible across the ligand and the receptor

 < 0.8 *radian *for 1 ≤ *i*, *j *≤ 3

(0.8 radian is approximately equivalent to 45.8 degrees)

**Figure 7:**
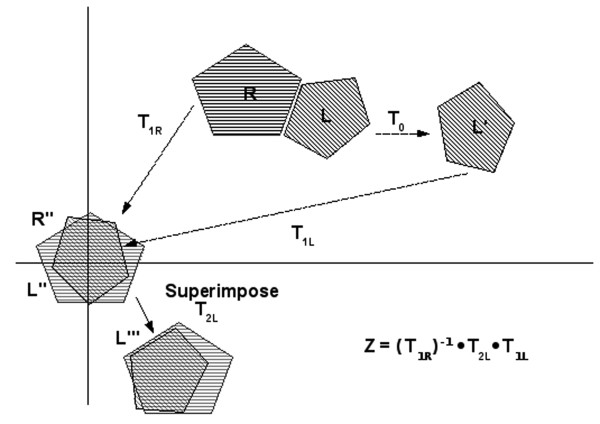
**Given a receptor R and a ligand L in their original orientation, a random orientation *T*_0 _is first applied to the ligand**. Selection of a three-point basis (two points + the average of the normals of the two points) for both the ligand (*L*" → *T*_IL_) and receptor (*T*_IR_) allows coordinates to be expressed in the new reference frame. Alignment of the matched set of coordinates in the hash space then produces an additional transformation (*L*"' → *T*_2*L*_). To move into the original coordinate system, the transformation matrix *Z*is applied to the ligand protein.

e) Compatibility of the angle between the normals and reference frame axes for the ligand and receptor.  < 0.8 radian for *i *= 1, 2 where  is the unit vector in the direction of *l*_1_*l*_2 _for the ligand and *r*_1_*r*_2 _for the receptor.

3. For all the receptor-ligand reference frames that exceed a threshold limit of votes (point pairs with matching labels). The threshold is set to 10 votes.

a) For each matching list of point pairs that exceed the minimum list of votes, calculate the alignment transformation that overlays these points [71].

b) Apply the transformation to the ligand protein. This transformation is actually a composition of three transforms as shown in Figure 7.

c) Calculate the number of clashes as indicated by the excluded volume and the extent of overlap given by the contact surface area. If the excluded volume exceeds the threshold value of 500 Å^3^, discard the transformation.

d) Output transformation and corresponding scores.

ii. Clear the voting list and proceed with the next reference frame.

5) For all candidate orientations, evaluate the weighted score based on Equations 6-8 described in the subsequent sections. Output the ranked list.

### Shape-based scoring function

The scoring function is based on geometric criteria and is composed of the following terms:

1) Term based on the orientation of the surface normals and the local shape correlation defined by the 3DZD. The term is further split into a reward and a penalty.

2) The buried surface area

3) The excluded volume

These individual terms are then combined with appropriate weighting factors (weight optimization performed using a genetic algorithm) to produce a single score with a higher value indicating a more favorable solution.

#### Term based on normals and 3DZD local shape correlation

This measure of the geometric surface complementarity is based on the orientation of the normals at the interface and the shape correlation between the 3DZD vectors. The score for a given ligand orientation is calculated using the following equation:

In the above equation, -1 ≤ *η*_*ij *_≤ 1 is the dot product of the normals while-1 ≤ *C*_*ij *_≤ 1 is the correlation between the 3DZD vectors as calculated by Eqn. 5. The score *ZN*(*η*_*ij*_, *C*_*ij*_) is computed for every point on the ligand protein *i *and its closest counterpart *j *on the receptor and summed up for a given docking conformation. The first part of the equation represents the reward score given to cases where a given pair *i *and *j *have normals with opposing angles (π radians, *i.e*. the scalar product is -1) and the 3DZD correlation close to 1 (*i.e*. perfectly complementary to each other). The maximum reward value of 1.0 is assigned to the term *A *when *C*_*ij *_= 1 and *η*_*ij *_= -1. The reward value is suitably reduced for cases where the correlation and the angle values are poor. The second part of the equation deals with penalization that occurs when the scalar product of the normals yields a positive value or if the correlation is negative (indicating a poor match between the shapes). The term *1-A *has a maximum value of 0.98 when *C*_*ij *_= -1 and *η*_*ij *_= 1. Both scores are weighted by an exponential distance term. The reward term and the penalty term is weighted separately with different weights (Table 5). Although these scores reflect the local shape complementarity, they do not adequately explain the extent of overlap of the surfaces and the resultant clashes that may occur due to the ligand orientation. Two additional terms have therefore been added to account for such cases.

#### Area of overlap

For a given orientation of the ligand, the amount of overlap is estimated by the buried surface area. The accessible area buried between the interacting components is given by:

Where *SASA *are the solvent accessible surface areas of the receptor R, the ligand_*L*_, and the complex_*RL*_, respectively. The areas were estimated using the boolean look-up tables approach by LeGrand and Merz [72]. Although larger values generally indicate a more stable association, they can often be misleading owing to the large interpenetration of the partners. A penalty term is also included to account for the steric clashes that may occur.

#### Excluded volume

Atom pairs at the interface that are closer than a cutoff distance (3 Å according to the CAPRI criteria [73]) are said to clash. Owing to the proximity, these clashing atoms have a repulsive effect which can be measured by the excluded volume term, for which an analytical expression has been proposed [74]. For two atoms, *a *and *b*, with van der Waals radii *R*_*a *_and *R*_*b*_, the distance between the atoms, *d*_*ab*_, the corresponding overlap volume *V*_*ab *_can be calculated as

where

The overall excluded volume is given by the sum of the volume overlaps over the receptor, *a *∈ *R*, and the ligand atoms, *b *∈ *L*:

#### Optimization of weights using Genetic Algorithm

Given the four scoring terms, the goal is to find a set of weighting factors *w*, *c*, such that the fitness function (Eqn. 12) is maximized [75]:

Here *s*_*i *_is individual scoring terms, *w*_*i *_is the set of weights for each term, and *c*_*i *_is the corresponding offset value. Eqn. 11 defines a ranking order on the set of docking predictions to be assessed. The form of Eqn. 11 was found to be superior to the linear weighting scheme [34] and has been duplicated in this study. A genetic algorithm (GA) based approach was used to determine an optimal set of weights and offset values for the terms. Starting with a population of randomly generated solutions, the GA iteratively attempts to improve the quality of the solutions using evolutionary schemes such as mutation and crossover with a suitably defined fitness function to guide the search.

The current implementation of the GA uses a(*μ*, *λ*) evolutionary strategy with self-adaptation [76] where the best *μ *individuals from a population of *λ *offspring are chosen as parents for the next generation. The fitness function is based on the Boltzmann-enhanced discrimination of receiver operating characteristic (BEDROC) [77]. The BEDROC metric is motivated by the fact that there are more false positives among the predictions and places more emphasis on hits located near the top of an ordered list as compared to those at the end. The metric has been shown to provide better discrimination of hits and non-hits in comparison to other measures such as average rank and the area under the ROC curve [78]. For a set of *N *predictions, with *n *hits, the BEDROC metric applies a continuously decreasing exponential weighting for the ranks of the hits and is calculated as:

Here *r*_*i *_is the rank of the hit and *β *= 160.9 is an exponent pre-factor defined such that 80% of the corresponding BEDROC score was based on the top-ranked 1% percent of the predictions. Given the discrepancies in ratio of hits to non-hits across different proteins, the average of the BEDROC values over the training set cases *i *was chosen as the final fitness.

Following is the algorithm flow for finding the weights based on GA:

1) Generate an initial population *P *= 30. Each individual in *P *is represented by 16 values, corresponding to the 4 feature weights, 4 offset values, and 8 mutation probabilities.

2) For a predefined number of generations *K *= 250 do

1. Create a new population using crossover and mutation. For each pair of parent individuals, two new offspring are created. Thus, for *n *= 30 individuals in *P*, *n*^2 ^= 900 offspring are created. The blend crossover (Eqn. 14) operator as implemented in OpenBeagle [79] was used. Given two parents  with mutation parameters , random values ,  and parameter *α *= 0.5 the children are recombined using the blend crossover operator as follows:

As different mutation strengths were used for each parameter, a Gaussian mutation operator [76] is applied (Eqn. 15). The update rule for mutation parameters *g*_*i *_and offspring *x*_*i *_for *β *= 0.01 and random values drawn from a Gaussian distribution (zero mean and unit variance) N(0,1) is given by

i. First the mutation probabilities are recombined (crossover) and then mutated.

ii. The weights and offset values are then recombined using the blend crossover operator, followed by mutation using the newly created set of mutation probabilities.

2. Assess the fitness of the newly created population

i. Use each individual set of weights (*w*_*i*_, *c*_*i*_) ∈ P to score and rank the sets of predictions for the roteins in the training set.

ii. Evaluate the BEDROC value (Eqn. 12) for each protein based on the ranks of the hits.

iii. Calculate the fitness for each individual as given by the average BEDROC value.

3. Select the *n *best set of parameters from the *n*^2 ^offspring. These will be the parents for the next generation.

3) At the end of *K *such cycles, record the best performing set of parameters for the current training set.

All the reported ranks for the LZerD predictions are based on the set of weights (Eqn. 11; Table 5) identified by the genetic algorithm (GA) trained on the data set listed in Table 2. The signs of the weights computed for the 3DZD-normal penalty, the buried surface area, and the excluded volume may seem counterintuitive *i.e*. they have positive, negative, and positive values when they are supposed to contribute as a penalty, a reward, and a penalty, respectively. However, they do function as desired, as for a vast majority of the cases, the computed terms are well below the offset values, thus effectively reversing the signs.

### Datasets and experimental setup

**Figure 8:**
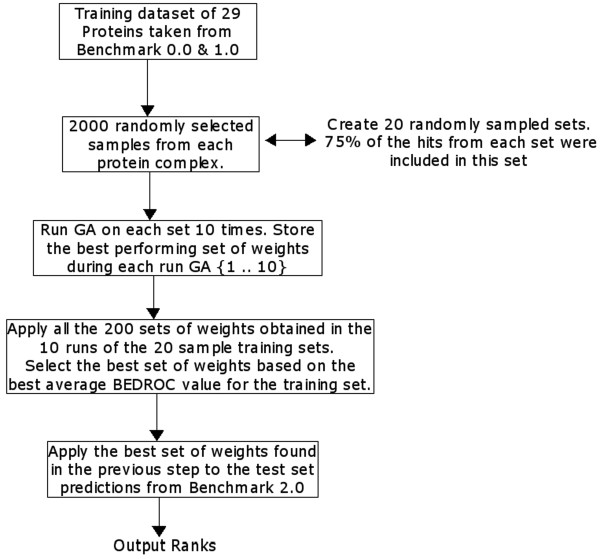
**Implementation of the training phase using the GA**. Twenty splits of the training data set are considered where each split is a collection of 2000 randomly selected predictions that include 75% of the hits from the generated data for each protein complex. Training is repeated 10 times on each split and the best set of weights from each split are recorded. The set of weights that gives the best average BEDROC on the entire training data set is applied to the test set predictions.

The training and testing procedure of the docking prediction is illustrated in Figure 8. The weighting factors (Eqn. 11) are determined based on a training set of 29 bound-bound protein complexes taken from ZDOCK benchmark datasets 0.0[80] and 1.0 [55]. For each of the 29 protein complexes in the training set, LZerD generated around 50,000-250,000 predictions, which were then classified as hits (predicted complex conformations with an interface RMSD ≤ 2.5 Å) and non-hits. Since the number of predictions was significantly high, the training set was split into 20 subsets each containing 2000 randomly chosen predictions for each protein complex. The subsets were chosen such that 75% of the hits for each complex were included for training. For each of the 20 sets the GA was run 10 times and the best set of weighting factors over each run was recorded. The 200 sets of values were then applied to the whole training dataset covering all predictions and the best performing set of weighting factors was chosen based on the one that yielded the largest average BEDROC score (Eqn. 12). The BEDROC score of the 200 training GA runs is provided as the Additional file 3.

The weighting factors obtained were then applied to a test set of (84 bound and unbound complexes) taken from the ZDOCK Benchmark 2.0 [21]. Protein complexes in ZDOCK Benchmark 2.0 that are common to the ZDOCK Benchmark 0.0 and 1.0 were removed from the training set thus ensuring an independent test set.

### Evaluation Criteria

All docking predictions were evaluated in terms of the interface (any atom within 10 Å of the other protein is considered to be part of the interface) RMSD between the bound and unbound interface C-*α *with values under 2.5 Å considered a hit[73]. In addition to the interface RMSDs, the ligand RMSDs (LRMSD) *i.e*. RMSD of the backbone atoms of the bound and unbound ligand proteins have also been calculated. According to the CAPRI criteria [73], LRMSD values less than 10 Å fall in the acceptable range. Another criterion, based on the mean of the logarithm of rank of the first hit[50] has also been used in the assessment

Here *N *is the number of complexes and *χ *refers to maximum value of the rank to be considered and is set to 1000. The value of *MLR *ranges between 1 (all rank 1 hits) to *χ *(no hits within the threshold).

## Availability and requirements

The docking program *LZerD *is written in C++. LZerD is freely available to academic institutions and Linux executables can be downloaded from our website, http://kiharalab.org/proteindocking/. The program requires a computer with at least 1.5 GB RAM operated by Linux OS. As the program requires some preprocessing of the PDB files, a shell script has been provided to automate the procedure. Thus, the user is only required to provide the ligand and receptor protein (PDB) files to be docked as input to the script. Output is in the form of PDB files of top ranking ligand orientations. The average times combining both docking and scoring range are about 1-2 hours for small proteins (about 400 points on the receptor and ligand) and it may take longer for larger proteins. This timing is reported on a computer with a dual-core 2.1 GHz processor with 8 GB RAM.

## Abbreviations

3D: three dimensional; 3DZD: 3D Zernike descriptors; LZerD: Local 3D Zernike descriptor-based Docking program.

## Authors' contributions

VV implemented the algorithms, designed and conducted the experiments, and drafted the paper. YDY participated in the development of geometric hashing. LS developed programs for computing the 3DZD and analyzed protein docking interfaces. DK conceived of the study, participated in its design and coordination, and helped to draft the manuscript. All authors read and approved the final manuscript.
